# Evaluation of Lee–Carter model to breast cancer mortality prediction in China and Pakistan

**DOI:** 10.3389/fonc.2023.1101249

**Published:** 2023-02-10

**Authors:** Sumaira Mubarik, Fang Wang, Lisha Luo, Kamal Hezam, Chuanhua Yu

**Affiliations:** ^1^ Department of Epidemiology and Biostatistics, School of Public Health, Wuhan University, Wuhan, China; ^2^ Department of Biostatistics, School of Public Health, Xuzhou Medical University, Xuzhou, Jiangsu, China; ^3^ Center for Evidence-Based and Translational Medicine, Zhongnan Hospital of Wuhan University, Wuhan, Hubei, China; ^4^ Nankai University, School of Medicine, Tianjin, China

**Keywords:** breast cancer, Lee-Carter model, forecast accuracy, life expectancy, MAPE

## Abstract

**Background:**

Precise breast cancer–related mortality forecasts are required for public health program and healthcare service planning. A number of stochastic model–based approaches for predicting mortality have been developed. The trends shown by mortality data from various diseases and countries are critical to the effectiveness of these models. This study illustrates the unconventional statistical method for estimating and predicting the mortality risk between the early-onset and screen-age/late-onset breast cancer population in China and Pakistan using the Lee–Carter model.

**Methods:**

Longitudinal death data for female breast cancer from 1990 to 2019 obtained from the Global Burden of Disease study database were used to compare statistical approach between early-onset (age group, 25–49 years) and screen-age/late-onset (age group, 50–84 years) population. We evaluated the model performance both within (training period, 1990–2010) and outside (test period, 2011–2019) data forecast accuracy using the different error measures and graphical analysis. Finally, using the Lee–Carter model, we predicted the general index for the time period (2011 to 2030) and derived corresponding life expectancy at birth for the female breast cancer population using life tables.

**Results:**

Study findings revealed that the Lee–Carter approach to predict breast cancer mortality rate outperformed in the screen-age/late-onset compared with that in the early-onset population in terms of goodness of fit and within and outside forecast accuracy check. Moreover, the trend in forecast error was decreasing gradually in the screen-age/late-onset compared with that in the early-onset breast cancer population in China and Pakistan. Furthermore, we observed that this approach had provided almost comparable results between the early-onset and screen-age/late-onset population in forecast accuracy for more varying mortality behavior over time like in Pakistan. Both the early-onset and screen-age/late-onset populations in Pakistan were expected to have an increase in breast cancer mortality by 2030. whereas, for China, it was expected to decrease in the early-onset population.

**Conclusion:**

The Lee–Carter model can be used to estimate breast cancer mortality and so to project future life expectancy at birth, especially in the screen-age/late-onset population. As a result, it is suggested that this approach may be useful and convenient for predicting cancer-related mortality even when epidemiological and demographic disease data sets are limited. According to model predictions for breast cancer mortality, improved health facilities for disease diagnosis, control, and prevention are required to reduce the disease’s future burden, particularly in less developed countries.

## Introduction

Cancer is one of the leading causes of death and disability worldwide. Breast cancer (BC) is the most common cancer diagnosed in women and is the first leading cause of cancer-related mortality in women (1, 2). It develops from a single cell that divides and multiplies into a lump that can be detected clinically. Its severe form from cancer’s prolonged development is the metastasis phase that is the more challenging treated phase (3, 4). The most common clinical manifestations of BC are a tumorous mass in the breast, enlarged lymph nodes in the armpits, and distant metastases. Recent studies have found that chronic inflammation plays a role in the development and progression of BC, in addition to genetics and the environment (5–7). Stage at diagnosis has been confirmed as a key prognostic factor for BC, and the previous study revealed that the advanced (III) and metastatic stage (IV) are highly associated with lower survival rates (8). Consequently, addressing healthcare policies for early diagnosis may reduce the morbidity and mortality of BC.

The burden of BC has been rising faster in low- and middle-income countries (LMICs) compared with high-income countries in last three decades due to the lack of healthcare policies. Drafting public health policy and devising interventions against cancer require accurate data in LMICs. However, because of insufficient and demographic and disease registration data in LMICs, statisticians are unable to evaluate disease consequences. Among the previous studies on BC mortality predictive models, some studies used simple models such as the joinpoint model or single-population model (9), and some have used machine learning algorithms to predict specific mortality for BC based on specific populations (10), but the application of dynamic predictions and models for whole population or age-specific mortality is still lacking. The introduction of stochastic mortality models provides us an opportunity to forecast cancer-specific mortality in LMICs. A number of suitable statistical approaches for mortality prediction have been proposed, and the performance of these models differs in various diseases and countries (11–13).

Several efforts have been directed toward finding an appropriate model for the accurate prediction of age-specific death patterns. In this regard, various parametric curves (14, 15) were considered to predict the mortality rate by year. Following these concepts, different approaches are established to predict mortality rates using stochastic models (16–19). As part of stochastic mortality models, the Lee–Carter (LC) method of mortality forecasting has become one of the most useful tools for forecasting age-specific mortality rates, and it has been previously employed for this purpose in several works (20–22). The model posits that variations in mortality trends over time are governed solely by a single parameter ( *k*_*t*_. )the mortality index. The mortality forecast is created using this index by selecting an appropriate time series model (23). LC-based modeling frameworks are one of the most efficient and transparent methods of modeling and projecting mortality dynamics (13, 16, 20, 24–29). Moreover, this model has also been suggested for predicting cause-specific mortality rate, for instance, BC causes mortality, which follows a smooth curvilinear and rapid change pattern over time (24).

Most Asian countries are facing an increased BC burden and do not have sufficient health-related facilities like proper diagnosis, screening, and treatment. Moreover, because of population aging and increasing life expectancy, the disease burden has been shifting from communicable to non-communicable diseases in these countries. These countries are having similar circumstances related to population expansion and aging (13). Furthermore, because of the shortcomings in these countries’ statistical registry systems, researchers are constantly confronted with the challenge of insufficient and unsatisfactory demographic and disease registration data sets to undertake suitable statistical analysis. Given the scarcity of data and its poor quality, advanced statistical approaches may be useful in modeling and predicting the mortality patterns in developing countries, and the LC model is one of the good options (11, 12).

Age-specific BC incidence curves have been shown to superimpose two distinct rate curves, one for early-onset BC with a median age of diagnosis below 50 years and another for late-onset BC with a median age of diagnosis above 70 years, disproving the long-held belief that the inflection point in the overall curve occurs around menopause (30, 31). Therefore, this study investigates the application of the LC model for BC mortality prediction between early-onset and age-screen/late-screen female populations in China and Pakistan. In our study, two age groups of 25–49 years and 50–84 years are stratified to assess the model applicability, and the early-onset population was defined as BC occurring in women under the age of 50, whereas the late-onset population was recognized as BC occurring in women aged 50–84 years. It is proved that early-onset BC has more aggressive clinicopathological characteristic and worse prognosis (32), so more specific studies are needed to compare the disparities of BC mortality trends between the early-onset and screen-age/late-onset female population. To the best our knowledge, this is the first study using advanced statistical methods in evaluating and predicting the BC-related mortality trends between the early-onset and screen-age/late-onset population for two developing countries.

## Data and methods

The annual mortality rates of the two Asian countries due to BC from 1990 to 2019 at the early-onset (age category of 25–49 years) and screen-age/late-onset (age category of 50–84 years) population were selected to run the application of the LC model. The Institute for Health Metrics and Evaluation (http://ghdx.healthdata.org/gbd-results-tool) provided BC mortality data for two Asian countries: China and Pakistan (33, 34). The availability of data and the sources are both included in the “Data and materials availability” declaration at the end of this study. BC mortality rates were calculated using the ratio of “number of deaths” to “exposure to risk”, which was grouped in a matrix for the specific age *x* and time *t*. We separated the data set into two parts to study the within-sample and out-of-sample model performance: training data set (1990–2010) and test data set (2011–2019). We fitted the) model on the training data set and evaluated the model performance using within and outside forecast accuracy.

The LC model (16) estimates mortality index *k*_*t*_. utilizing age-specific death rates. This assessment is made for the early-onset and screen-age/late-onset female population for China and Pakistan. The estimated model is evaluated for both goodness of fit and accuracy of forecast ability. Using the mortality index ( *k*_*t*_. )stimation, BC death rates and life expectancy may be predicted.

## Statistical analysis

### Lee–Carter model

The LC model considers a statistical and demographic model that predicts mortality rates to derive life tables (16). The fundamental assumption of the model is that there is a linear connection between the age-specific death rates on logarithm scale ( *m*_*x*,*t*_). age interval *x* and time *t*. This relationship is described as follows:


(1) $$m_{x,t}=exp\left(a_{x}+b_{x}k_{t}+e_{xt}\right),\;\;\;\;t=1,2,\ldots ,n\;\;x=1,2,\ldots ,\omega$$

Equation (1) can be expressed by taking natural logarithm on both sides as follows:


(2) $$f_{x,t}=\ln\left(m_{x,t}\right)=a_{x}+b_{x}k_{t}+e_{xt},\;\;\;\;t=1,2,\ldots ,n\;\;x=1,2,\ldots ,\omega$$

In Equation (2), *m*_*x*,*t*_. represents age-specific death rate for the *x* age interval and year *t*, *a*_*x*_. notes the average age-specific mortality, *k*_*t*_ represents the mortality index in the year t, *b*_*x*_. a mortality deviation caused by changes in the *k*_*t*_. index, *e*_*xt*_ is the random error, and *ω*. the start of the last age interval (35).

There are various issues with parameter estimation when the bilinear term *b*_*x*_ *k*_*t*_ is present. Lee and Carter used a technique known as the singular value decomposition (SVD) to partially alleviate these issues. This method necessitates the assumption that the random component is homoscedastic. According to research, the sample’s variance is not distributed uniformly (36, 37). For instance, when contrasting the variance between the age ranges of 25–50 years and 50+ years, this phenomena is very obvious. The greatest likelihood method is an alternative to the SVD approach. We assume that the number of deaths is a random variable with a Poisson distribution while using this estimation technique.

The earlier research demonstrates that mortality modeling can be done successfully using the LC models. To estimate structural parameters, one can utilize the greatest likelihood technique. However, when simulating the number of deaths, additional distributions in addition to the Poisson distribution should be utilized. Previous studies have demonstrated that using the negative binomial distribution can produce positive outcomes when dealing with heterogeneous populations. In that instance, the LC model offered better results in terms of goodness of fit (36).

To get an estimate for the values of *a*_*x*_, *b*_*x*_ and *k*_*t*_, a system of simultaneous equations is needed to be solved, which is called the system’s solutions. Therefore, death rates for various age groups (*r*) observed at different points in time (*n*) produces a system of equations containing 2*r*+*n* unknown variables that correspond to the total of the *r* values of *a*_*x*_, r values of *b*_*x*_, *n* values of *k*_*t*_, and the total number of equations is *r*×*n*. The matrix form of such system of equations can be represented as below:


(3) D=A+b.k

D is an matrix of the order *r*×*n*, and an element D_i, j_ represents the age-specific death rate (on natural logarithm scale) in the age group i in year j. A denotes a matrix with of order *r*×*n*. For the same year j, the elements that belong to the same categories are identical: a_ij_=a_2j_=...a_rj_, while b represents a vector of order *r*×1 and k is a vector of order 1×*n*

A unique solution of equation (3) can be arrived by imposing following two restrictions: $\sum_{x=1}^{\omega }b_{x}=1;\;\;\;\;\;\;\;\sum_{t=1}^{n}k_{t}=0$ .

When such restrictions are applied, the *a*_*x*_ coefficient represents mean mortality rate over time. Therefore, the parameter *b*_*x*_ and *k*_*t*_ are calculated individually. The coefficients of *a*_*x*_ are obtained from the following equation.


(4) $$a_{x}=\frac{\sum_{t=1}^{n}\ln\left(m_{x,t}\right)}{n}$$

When the matrix *A* is computed, the system (3) may be recast as follows:


(5) $$D^{*}=D-A=b.k$$

The aforementioned system offers a unique solution when these restrictions are met. The SVD technique is used to estimate the *b* and *k* parameters. This technique is used to get the best fit of least squares. D* can be expressed as the product of two matrices using SVD. The element (*i*, *j*) in D* shows the product of the *i* ^th^ row of *B* and the *j* ^th^ row of *K*, resulting in the following:


(6) $$m_{i,j}=\sum_{l=1}^{r}B_{i,l}K_{\;j,l}^{T}$$

As a result, the decomposition yields *r* terms that exactly match the D* matrix element. Lee and Carter (16) proposed D* as the product of the *b* and *k* vectors. When employing SVD, these were regarded first-order approximations, i.e., D′ can be represented as follows:


(7) $$D^{\prime }\approx B_{1}K_{\;1}^{T}$$

Finally, *B*_1_=*B* and *K*_1_=*K* are computed, implying an initial estimate of the model’s parameters in equation (14).

### Re-estimation of *k*_*t*_ parameter

In general, the results produced from the model’s initial estimates do not offer an acceptable match to the observed data. Lee and Carter (16) and Bell (38) point out that there may be deviations from the predictions. Therefore, a second step is required to estimate the parameters. This step utilizes the *a*_*x*_ and *b*_*x*_ values from the previous step to get a new estimate of *k*_*t*_ reflecting that a total number of deaths for the given year must be observed. The goal is to determine *k*_*t*_ values, which satisfy the following condition:


(8) $$D_{t}=\sum_{x=0}^{\omega }N_{x,\;t\;}exp(a_{x}+b_{x}k_{t}+e{x,\;t}_{)}$$

In Equation (8), *D*_*t*_ is the total number of deaths during the calendar year *t*. The population in the *x* age interval in the year *t* is denoted by *N*_*x*, *t*_ and ω is the age of the final observed group in mortality tables (16). The model estimation is carried out using the ilc package in R programming language (Development Core Team, 2008).

### Age-specific death rate prediction

After obtaining the time series for the *k*_*t*_ index as described in section (2, 3), autoregressive integrated moving average (ARIMA) model may be used to forecast such an index; then, it is possible to obtain the death rates for the anticipated years. In the equation, the predicted values of *k*_*n*+*h*_ e substituted.


(9) $${\hat{m}}_{x,\;n+h}={\hat{m}}_{x,\;n}\;exp\left\{{\hat{b}}_{x}\left({\hat{k}}_{n+h}-{\hat{k}}_{n}\right)\right\},\;h=1,2,\ldots \;x=1,2,\ldots ,\;\omega$$

In Equation (9), *n* represents the most recent year for which data are available, *h* represents the prediction horizon, and *x* represents the age group. Equation (9) is used to forecast death rates based on the most recent death rate. To anticipate death rates, the LC model offered an approximate prediction interval (16). The interval is calculated using estimates of *b*_*x*_ pameters and standard errors of the *k*_*t*_ projections.


(10) $$PI:\left\{m_{x,t}\;exp\left(2b_{x}\;se_{kt}\right)\right\};\;\left\{m_{x,t}\;exp\left(-2b_{x}\;se_{kt}\right)\right\}$$

### Life expectancy at birth

Age-specific life expectancy estimates the average number of years left in a person’s life, assuming that current mortality rates remain unchanged. It is computed by considering age-specific death rates (39). The standard technique of Chiang (40) is used to calculate life expectancy at birth using projected death rates. The life expectancy at x, *e*_*x*_., is stated as follows:


(11) $$e_{x}=\frac{T_{x}}{l_{x}}$$

*T_x_* presents the total number of years that the cohort has lived during the age interval and subsequent age intervals, and *l*_*x*_ denotes number of individuals alive at the start of the *x* age interval from a population of *l*_0_ newborn infants. This is generally expressed as *l*_0_ =100,000 (23).

### Error measure

The predictive ability of the model was evaluated by mean absolute percent error (MAPE), using the following formula:


$$MAPE=\left(\frac{1}{H}\sum_{h=1}^{H}\left|e_{t+h}\right|\right)\times 100\;$$

where $e_{t+h}=\frac{\text{actul}\;\text{value}-\text{predicted}\;\text{value}}{\text{actual}\;\text{value}}$ , and H denotes the number of predicted sample size.

To assess the forecast ability of the model, both within-sample and out-of-sample forecast accuracy were tested. A model is deemed to be well-fit if it delivers a strong fit within-sample to the historical data and good out-of-sample forecasts. As a result, out-of-sample predictive accuracy was investigated to confirm the model’s predictive accuracy with consistency. The following steps were taken into account when evaluating forecast accuracy. To begin, we must select the metric of interest, which includes the anticipated variable. Forecasted variable measurements could include death rates, life expectancy, or future survival rates. As this study aims to examine the feasibility of stochastic mortality model on BC mortality data, therefore, we focused on BC mortality rates. We forecasted BC mortality rates from 2011 to 2019 using the fitted model and calculated life expectancy by comparing forecasts with the actual values.

## Results

### Breast cancer mortality behavior

**Figure 1:**
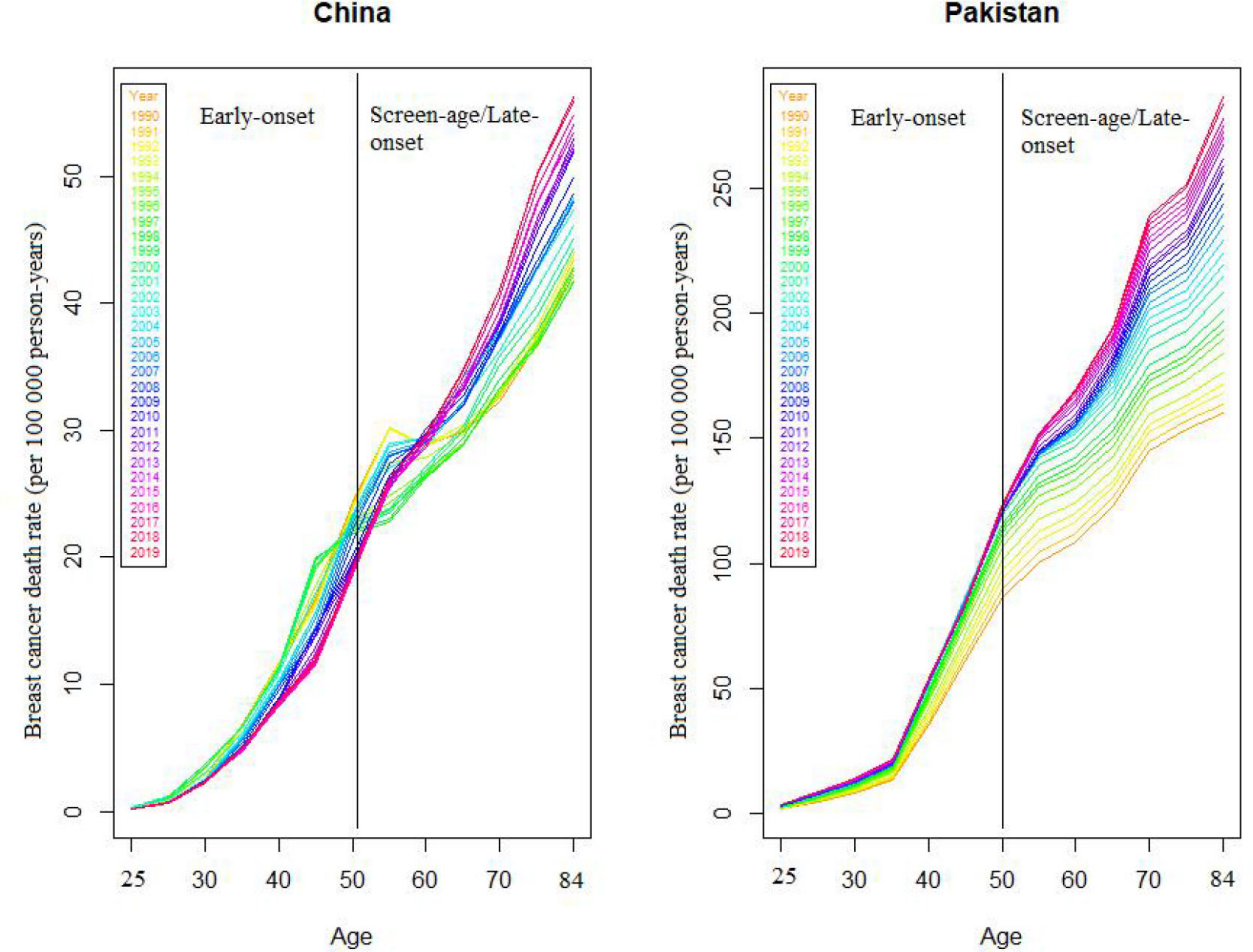
Death rates (per 100,000) due to female breast cancer in China and Pakistan, 1990–2019.

We found that BC mortality has gradually grown with time when we examined the variations in BC mortality rates related to both age *x* and period *t*. **Figure 1** depicts the general patterns in BC mortality rates from 1990 to 2019 for two countries to investigate this process. We may also see that death trends are not consistent between ages and throughout time. In both countries, there is an increasing disparity among older age groups (>50 years), particularly around the age of 84 years.

### Model estimation

**Figure 2:**
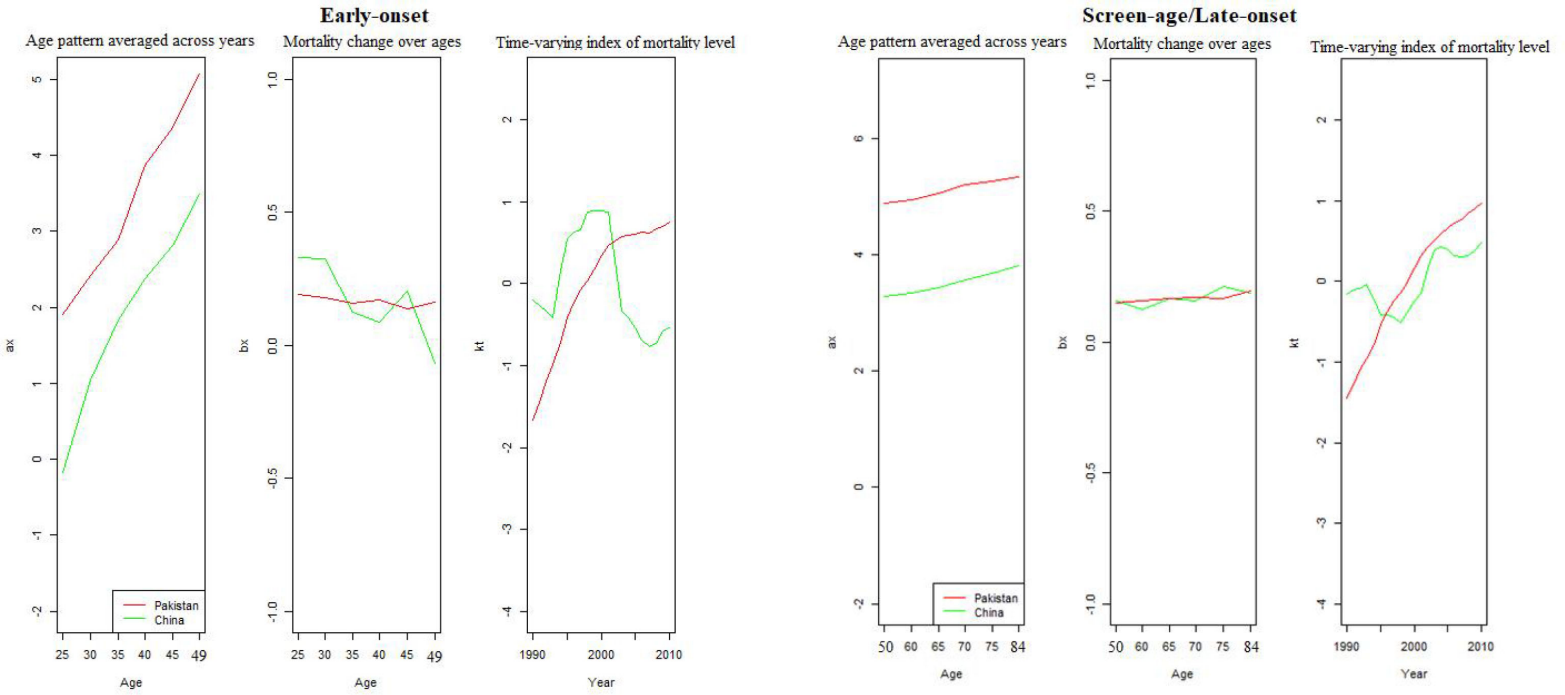
Model estimation between the early-onset and screen-age/late-onset population for China and Pakistan.

To assess the model’s within-sample and out-of-sample performance, we modified the model by removing the last 9 years of data from both countries’ data sets. Fitting the stochastic mortality model (LC) for both the early-onset and screen-age/late-onset population is the initial stage in the analytical process. **Figure 2** shows the estimated parameters of the LC model for China and Pakistan for both the early-onset and screen-age/late-onset population. The model’s percentage of variation (PV) was around 86% and 89% between the early-onset and screen-age/late-onset population for the China, and 98% for both the early-onset and screen-age/late-onset population for Pakistan. The variation in PV between two countries’ data sets is caused by BC mortality patterns and various data features, as shown in **Figure 1**. We could show that the BC mortality rates at older ages were less consistent in Pakistani data than in China; as a result, the LC model fit the Pakistan data better and explained the higher PV in the screen-age/late-onset population than in China.

**Figure 3:**
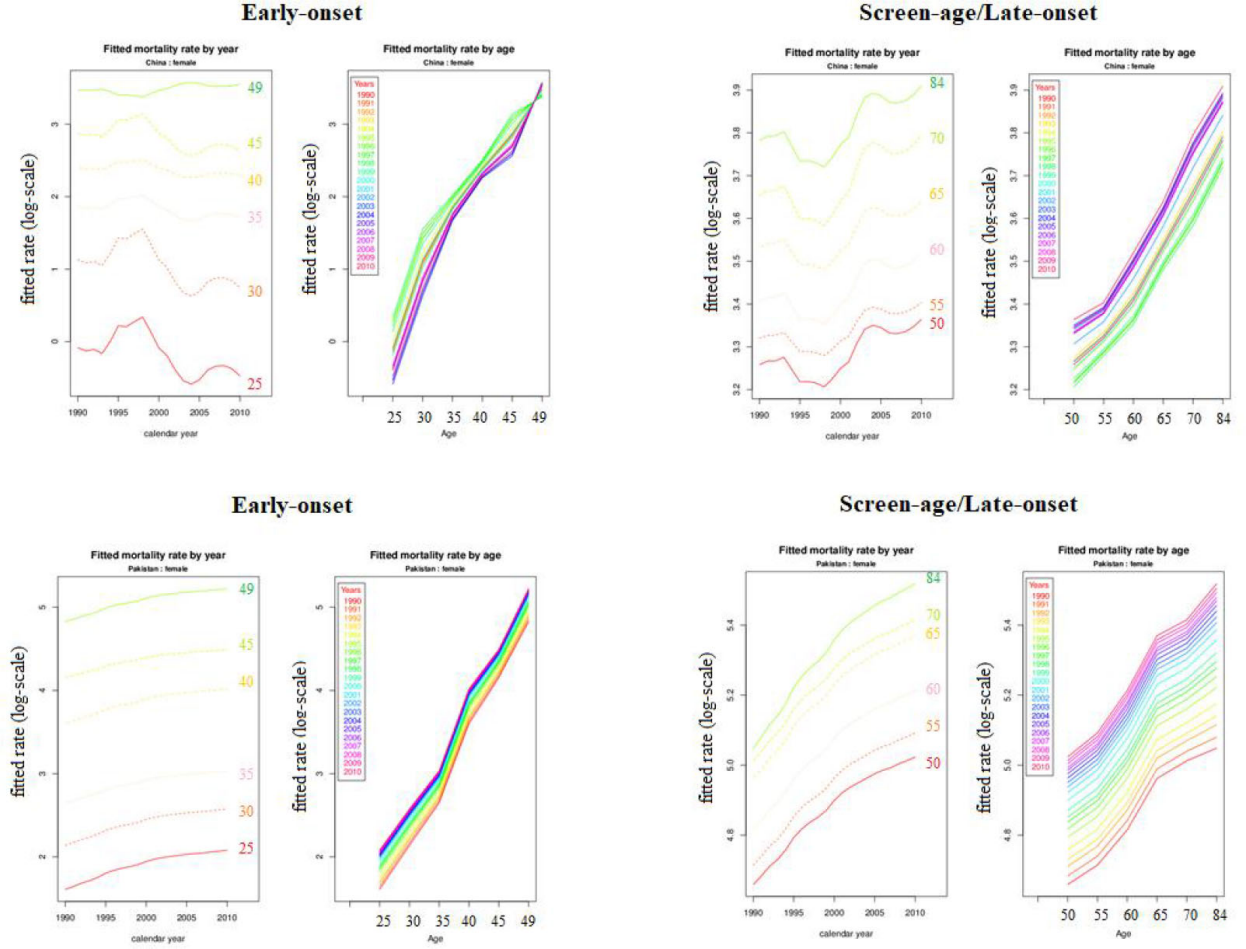
Fitted breast cancer mortality rate (log-scale) between the early-onset and screen-age/late-onset population for China and Pakistan.

We can observe that the variance trend ( *b*_*x*_) among screen-age/late-onset population is gradually increasing with age for both China and Pakistan, whereas, over time (*k_t_*), these mortality differences are steadily growing after 2000; particularly, these differences were higher for Pakistan than that for China (**Figure 2**). Moreover, the fitted BC mortality rates by age and year through the LC model for both the early-onset and screen-age/late-onset population for China and Pakistan are depicted in **Figure 3**.

### Model evaluation and forecasting

**Figure 4:**
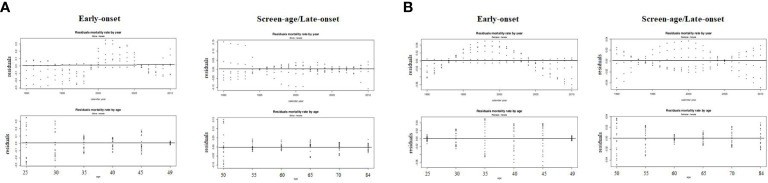
Residuals mortality rates by age and year from the LC model between the early-onset and screen-age/late-onset population in **(A)** China and **(B)** Pakistan.

When the residuals are independent and identically distributed, a matching fit is seen. To validate this condition, the fitted model’s residual death rates by age and year were calculated (**Figure 4**). In the screen-age/late-onset population, residual death rates by age and years were predicted to be more consistent. In Pakistan, these errors were lower than in China. Furthermore, error estimates were produced to confirm the error disparities across different population models, as shown in **Table 1**. By evaluating the error between the early-onset and screen-age/late-onset population, we noticed that the error measures for screen-age/late-onset model are smaller than the early-onset model. Between China and Pakistan, these errors were lower in the Pakistan’s data set compared with that in China (**Table 1**).

**Table 1:** Error measures from fitted Lee–Carter model of the early-onset and screen-age/late-onset breast cancer population for China and Pakistan.

	China		Pakistan	
	Early-onset	Screen-age/late-onset	Early-onset	Screen-age/late-onset
**Averages across ages**				
MPE	0.01416	0.00135	0.00039	0.00029
MAPE	0.10212	0.02437	0.01891	0.01371
**Averages across years**				
IPE	0.34702	0.02743	0.01139	0.0063
IAPE	2.49338	0.51237	0.54902	0.30285

MPE, mean percent error; MAPE, mean absolute percent error; IPE, integrated percent error; IAPE, integrated absolute percent error.

**Figure 5:**
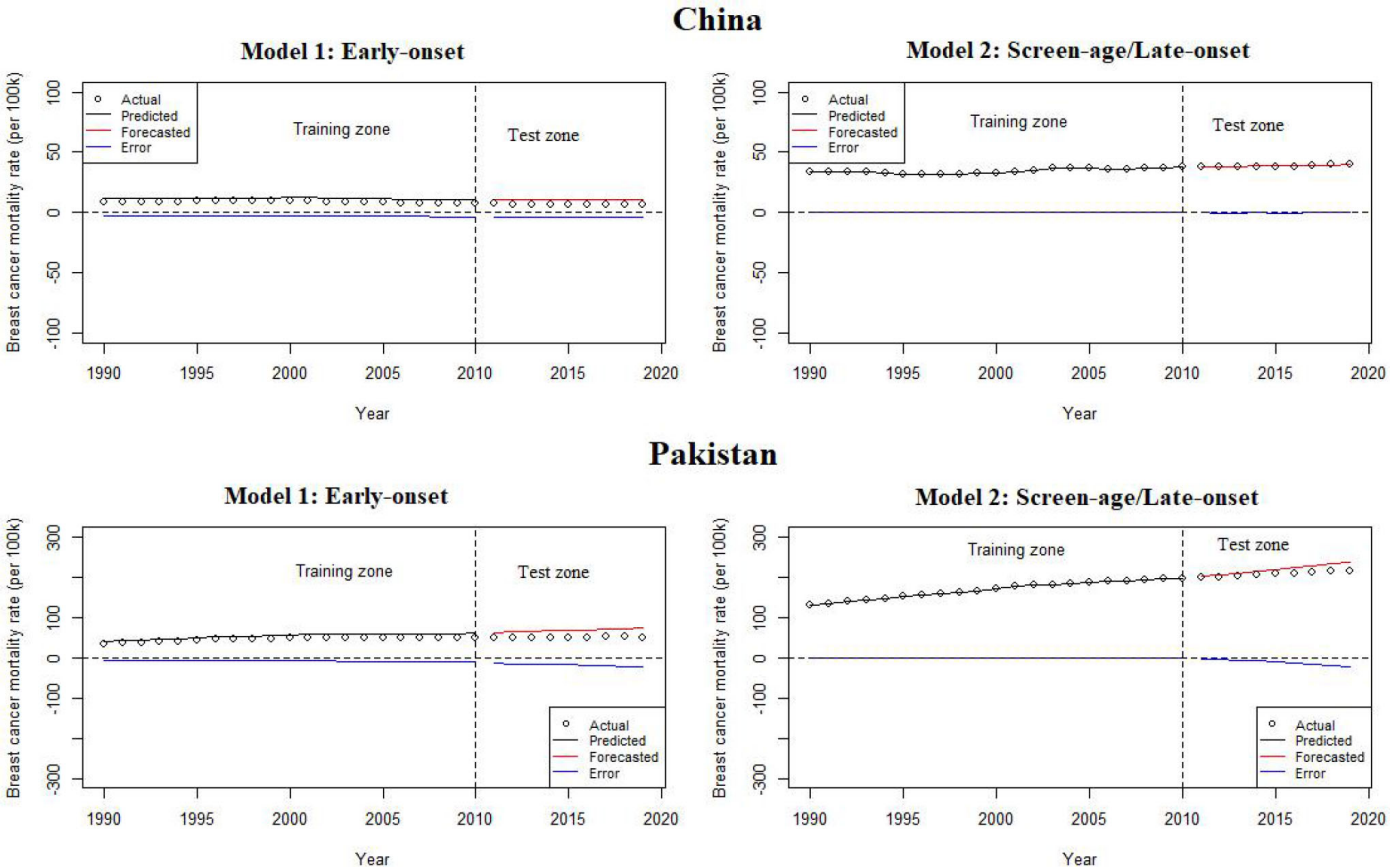
Lee–Carter model predicting ability between the early-onset and screen-age/late-onset population in China and Pakistan.

**Figure 6:**
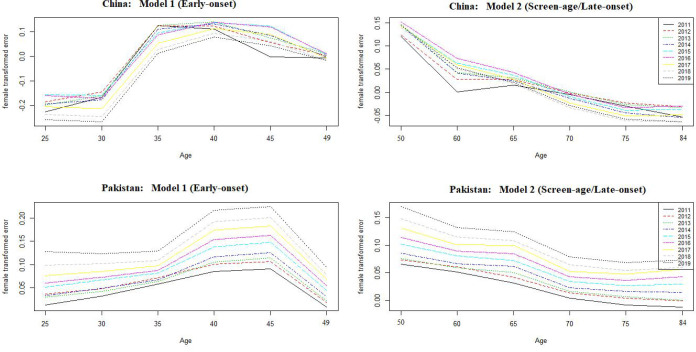
Forecast error over ages between the early-onset and screen-age/late-onset population in China and Pakistan.

Forecasts were calculated in our study on the basis of the evolution of time parameter ( *k*_*t*_); and errors in age parameters (*a*_*x*_ and *b*_*x*_) were not considered because, according to the literature, the standard errors of (*a*_*x*_) and (*b*_*x*_) become less significant over forecast time in comparison to the standard error of parameter ( *k*_*t*_) (16). The model predicting ability for both the early-onset and screen-age/late-onset population for China and Pakistan is shown in **Figure 5**. Overall, we observe that the prediction error for the screen-age/late-onset model was lower than that for the early-onset model for both China and Pakistan. Furthermore, we observed that the LC approach has provided almost comparable results between the early-onset and screen-age/late-onset populations in forecasting accuracy for less invariant mortality behavior over time like in Pakistan (**Figure 5**). Moreover, the trend in forecast error (test data set) was gradually decreased in the screen-age/late-onset BC population than early-onset for both China and Pakistan (**Figure 6**).

**Figure 7:**
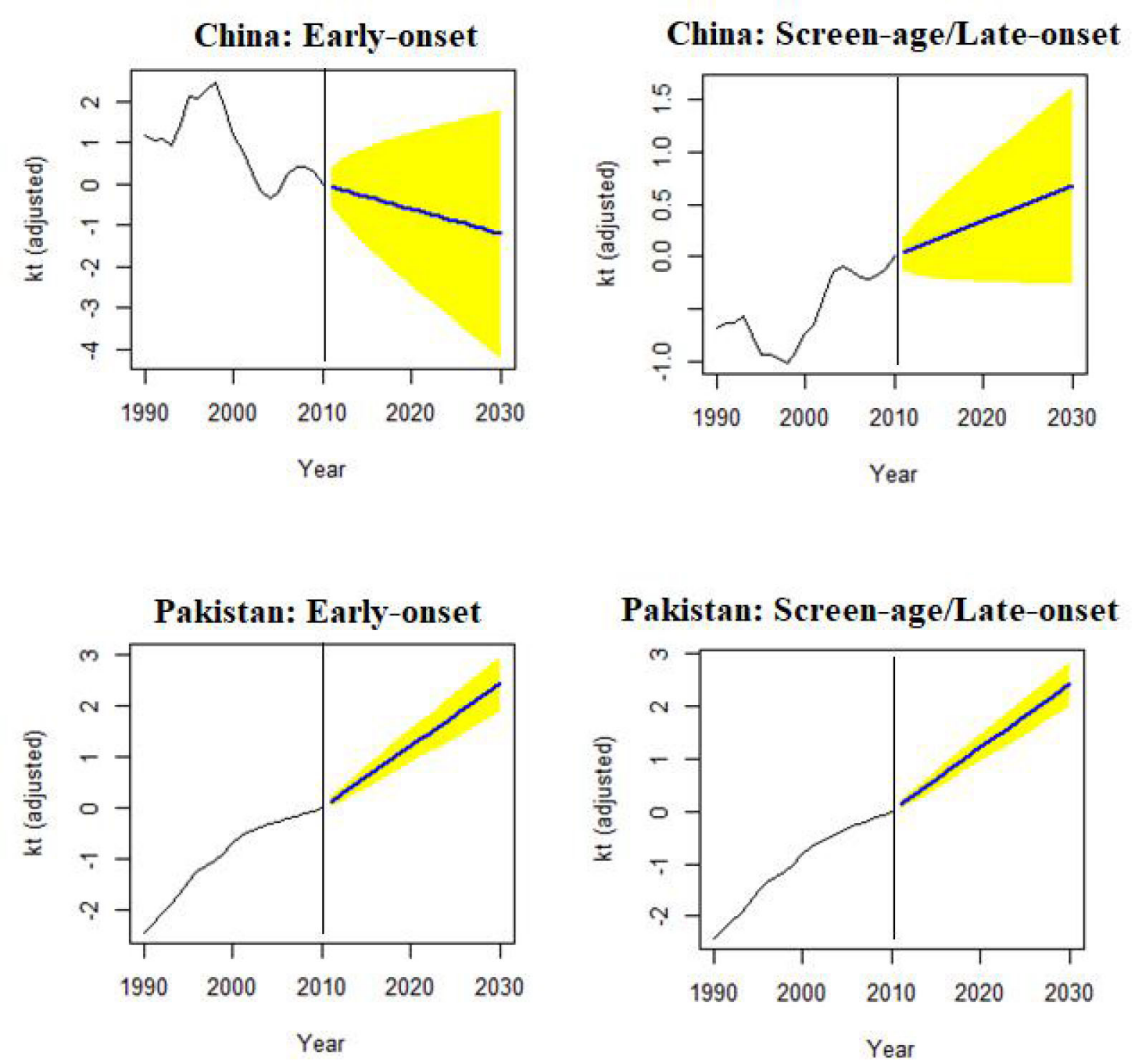
Forecast of mortality index (*k_t_*) to 2030, in the early-onset and screen-age/late-onset female breast cancer population for China and Pakistan.

To confirm the out-of-sample forecast accuracy, we also looked at the mean and variance of life expectancy forecast errors over the projected period. **Table 2** demonstrates the minimum variance of life expectancy forecast error for both countries’ screen-age/late-onset populations. Finally, according to the model prediction, the BC mortality was predicted to increase by 2030 for both the early-onset and screen-age/late-onset population in Pakistan, whereas, for China, it was expected to decrease in early-onset population (**Figure 7**).

**Table 2:** Mean and variance of forecast error in life expectancy derived from the Lee–Carter model.

Country	Early-onset		Screen-age/late-onset	
	Mean	Variance	Mean	Variance
China	0.034	0.006	0.020	0.0012
Pakistan	0.033	0.004	0.013	0.0010

## Discussion

This study presented the application and evaluation of the LC model on age-specific BC death rates between the early-onset and screen-age/late-onset female populations in China and Pakistan for the period 1990–2019. We separated the data set into two parts to study the within-sample and out-of-sample model performance: training data set (1990–2010) and test data set (2011–2019). We test the model on the training data set and assessed its performance using within and outside forecast accuracy. The index of the level of BC mortality between the early-onset and screen-age/late-onset population as well as and shape and sensitivity coefficient by age were found through this approach. The mortality rates for the period 2020 to 2030 were predicted using the ARIMA model between the early-onset and screen-age/late-onset in the female population for each country under study, and it is necessary to highlight that the period under this study represents the maximum period of data availability. The LC approach presented in this study provides the adequate fit on BC mortality data between the early-onset and screen-age/late-onset female populations for China and Pakistan. However, there were some differences in forecast accuracy measure between the early-onset and screen-age/late-onset population, where we have observed the most accurate fit and strong predictive ability of model for screen-age/late-onset population for both countries. The reason might be the more smoothing mortality behavior in this population as compared to the early-onset. In some the previous studies, the LC approach has been suggested for mortality prediction among older populations (13).

According to the recent estimation of Global Burden of Disease GBD, among women, BC caused the most disability-adjusted life years, deaths, and years lived with disability (41). The differences in age-specific BC mortality between the early-onset and screen-age/late-onset female population in China and Pakistan followed a smooth function with minor observational error. Our findings showed that BC has a high variance in older age groups, where the population is lesser, and, among younger age group too, the mortality rates were low. These findings are consistent with the previous studies, which revealed considerable variability in rates based on geography and age group, notably for mortality rates (42, 43). A related study found a similar pattern in US mortality statistics, where statisticians discovered that age-specific mortality was higher than 1.0/100,000 for very small populations (44). Stochastic mortality models represent forecasting mortality trend based on such data pattern, and these approaches have been applied in various studies in different countries for all-cause and cause-specific mortality prediction (28, 44–46).

The general mortality index (*k_t_*) is a time series analysis representing the variability over time. It shows a declining trend in BC mortality for the early-onset Chinese population and increasing trend for the screen-age/late-onset population in both China and Pakistan. The plausible reasons for the predicted decline in BC mortality are not yet clear and demand more research. Proper health infrastructure and therapies availability might explain some portion of predicted reductions in China among the young population. This method increases early detection while also providing efficient treatment. Most women under the age of 50 who work in cities have access to employer-sponsored services such as medical exams and free breast ultrasounds once or twice a year. Previous research has demonstrated that an ultrasound is performed before to Chinese women’s mammography to prevent and control BC (47). Mubarik et al. (2020) analyzed the trends and forecasts in BC mortality and predicted greater BC mortality rates among older populations in numerous Asian countries, including Pakistan, in 2030 (13). The rising behaviors in the patterns of BC mortality might be due to lack of BC early screening, diagnosis, and treatment regime, as compared with developed countries (13). The proposed model for risk factors and their roles in triggering BC therapy may be used in future studies to improve healthcare tactics targeting this disease.

This study presents the application and evaluation of the Lee and Carter’s approach for BC mortality prediction. As the LC method appears to be a method with probabilistic support, this strategy generates many measurements and outcomes that characterize current and future patterns in BC mortality. As in many other countries, the use of this strategy in China and Pakistan produced better outcomes in terms of least forecast error and diagnostic measures. It is important to note that the study duration is significantly shorter than those of Sweden, the United States, and Chile (16, 35, 48). These three investigations covered time spans of more than 100 years. The amount of projections that can be generated is affected by the time period under consideration. Because the LC model is entirely reliant on historical mortality and population statistics, it is critical to have solid data over a long period of time. This demonstrates the significance of obtaining data efficiently and keeping records up to date in a certain region, country, or sub-national level.

This study has some strengths. First of all, our study examined the applicability of the multi-population random mortality models, the LC dynamic mortality assessment model, in the prediction of BC mortality in China and Pakistan. The LC model is considered as one of the most representative dynamic models in the random prediction methods, but, as far as we know, this is the first time to verify the statistical model of BC mortality prediction in two developing countries. In addition, we further compared the differences in mortality trends of BC between the early-onset and screen-age/late-onset population and verified that the model was more accurate in predicting age/late onset group, filling the gap in this regard. Similarly, this study has some limitations. First, we conducted our analysis based on secondary data; therefore, the accuracy of the model simulation is limited by the accuracy of GBD estimates. Second, we did not consider other covariates that may affect the risk of death from BC in the two countries in the model evaluation, such as health policies and treatment conditions. Third, our model was trained and tested for different parts of the same data set, and the actual effect may not be as good as the alternative, which is to train on one data set and validated on the other data set, so that the external validation is more able to demonstrate the generality of the model. As, for validation, our work made use of a comparable data set. If screening, diagnostic, and treatment methods change between different centers and over time, further analysis using an independent data set would be helpful to assure adaptability.

## Conclusion

The LC model can be considered to forecast BC mortality to project the future life expectancy at birth, particularly among the screen-age/late-onset population. By model prediction, BC mortality is expected to increase to 2030 for both the early-onset and screen-age/late-onset population in Pakistan. In China, it is likely to decrease for the early-onset population. Hence, this approach may be helpful and convenient to predict the cancer related mortality even for insufficient epidemiological and demographic disease data set. According to model prediction to BC mortality, better health facilities in terms of disease diagnosis, control, and prevention are needed to minimize this disease’s future burden, particularly in less developing countries.

## Data availability statement

Publicly available datasets were analyzed in this study. This data can be found here: The dataset analyzed during the current study are available in the Institute for Health Metrics and Evaluation (IHME): http://ghdx.healthdata.org/gbd-results-tool.

## Author contributions

CY supervised the study. SM and CY conceptualized the analysis. SM did the data analysis and wrote the first draft of the paper. FW, LL, and KH reviewed and provided comments on the first draft. All authors reviewed and approved the final manuscript.
